# TAFPred: Torsion Angle Fluctuations Prediction from Protein Sequences

**DOI:** 10.3390/biology12071020

**Published:** 2023-07-19

**Authors:** Md Wasi Ul Kabir, Duaa Mohammad Alawad, Avdesh Mishra, Md Tamjidul Hoque

**Affiliations:** 1 Computer Science Department, University of New Orleans, New Orleans, LA 70148, USA; mkabir3@uno.edu (M.W.U.K.); dmalawad@uno.edu (D.M.A.); 2 Department of Electrical Engineering and Computer Science, Texas A&M University-Kingsville, Kingsville, TX 78363, USA; avdesh.mishra@tamuk.edu

**Keywords:** backbone torsion angle, torsion angle fluctuations, machine learning

## Abstract

**Simple Summary:**

This study aimed to create an intelligent computer model called TAFPred to predict how proteins move and twist by looking at their sequences. By analyzing different features of the protein sequences, the model can accurately estimate the degree of flexibility of protein structures per residue. The investigators used an advanced machine learning technique called LightGBM to make these predictions even better. Compared to existing methods, TAFPred significantly improved in accurately predicting how proteins bend and twist within the individual and collective residual degree of freedom. This study is vital because understanding protein flexibility helps us know how they function in our bodies. By improving our ability to predict protein movements, this study brings us closer to unlocking the secrets of how proteins work and the role of protein flexibility in cellular functions, which can have critical applications in medicine and biology.

**Abstract:**

Protein molecules show varying degrees of flexibility throughout their three-dimensional structures. The flexibility is determined by the fluctuations in torsion angles, specifically phi (φ) and psi (ψ), which define the protein backbone. These angle fluctuations are derived from variations in backbone torsion angles observed in different models. By analyzing the fluctuations in Cartesian coordinate space, we can understand the structural flexibility of proteins. Predicting torsion angle fluctuations is valuable for determining protein function and structure when these angles act as constraints. In this study, a machine learning method called TAFPred is developed to predict torsion angle fluctuations using protein sequences directly. The method incorporates various features, such as disorder probability, position-specific scoring matrix profiles, secondary structure probabilities, and more. TAFPred, employing an optimized Light Gradient Boosting Machine Regressor (LightGBM), achieved high accuracy with correlation coefficients of 0.746 and 0.737 and mean absolute errors of 0.114 and 0.123 for the φ and ψ angles, respectively. Compared to the state-of-the-art method, TAFPred demonstrated significant improvements of 10.08% in MAE and 24.83% in PCC for the phi angle and 9.93% in MAE, and 22.37% in PCC for the psi angle.

## 1. Introduction

**Figure 1:**
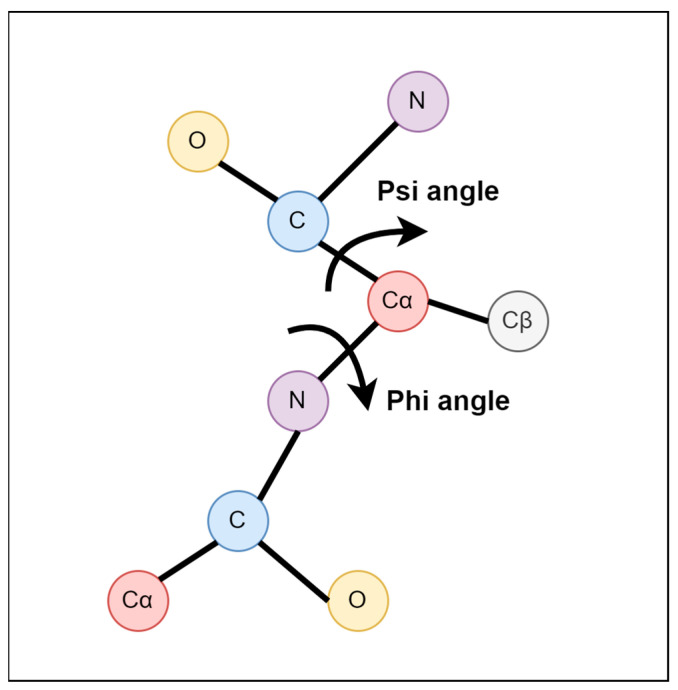
Torsion angles phi (φ) and psi (ψ). The phi angle is the angle around the -N-CA- bond (where ‘CA’ is the alpha-carbon), and the psi angle is the angle around the -CA-C- bond.

Proteins are organic molecules composed of carbon, hydrogen, nitrogen, oxygen, and sulfur [1,2,3,4,5]. The core carbon atom is coupled to a side chain group, an amine group, a carbonyl group, and a hydrogen atom [6] to form a protein molecule. Protein molecules are essential and comprise many structures and functions within the cell. They also play an important role in the cell, creating structures and performing numerous functions [7]. Protein molecules, such as actin and tubulin, can serve as structural and functional entities, such as enzymes that facilitate vital metabolic reactions. The tertiary structure of a protein refers to its spatial folding in three dimensions. Following ribosome-mediated synthesis, the polypeptide chain may require the assistance of chaperone proteins [8,9]. These chaperones also establish temporary hydrogen bonds with the polypeptide chain, guiding it to the correct conformation. This process ensures proper folding, leading to the protein’s appropriate functionality. Protein structure can be illustrated by backbone torsion angles (Figure 1): rotational angles about the N-Cα bond (φ) and the Cα-C bond (ψ) or the angle between Cαi-1-Cαi-Cαi + 1 (θ) and the rotational angle about the Cαi-Cαi + 1 bond (τ) [10]. Prediction of the Cα atom-based angle has demonstrated their potential usefulness in model quality assessment and structure prediction [11,12].

Proteins are not static structures. They undergo conformational changes as part of their function [3,13]. This might involve moving to bind to another molecule, changing shape to carry out a catalytic function, or flexing to allow the passage of another molecule [5,14]. Some protein molecules do not fold to their native state and remain in a flexible state [15]. Torsion angle fluctuations, often referred to as changes in the dihedral angles along the protein backbone, are critical to understanding protein dynamics and function in structural biology [16]. These fluctuations often indicate the flexibility or rigidity of different regions in the protein structure, giving insights into the dynamics and conformational changes that proteins undergo to fulfill their functions. Understanding the range and frequency of these torsional fluctuations helps predict the protein’s functional states [16]. Changes in torsion angles guide the process by which a protein folds from a linear chain of amino acids into its functional three-dimensional structure. By studying these changes, researchers can gain insights into the protein folding process, which is crucial for understanding diseases related to protein misfolding, like Alzheimer’s and Parkinson’s [17,18]. Fluctuations in torsion angles can also affect how a protein interacts with other molecules, such as drugs, substrates, or other proteins. Understanding these dynamics can guide the design of drugs that can effectively bind to a protein and modulate its activity [17]. Moreover, torsion angle changes can propagate through a protein structure leading to allosteric effects, where binding at one site affects the protein’s behavior at a distant site. Understanding these effects is crucial for developing drugs that can modulate protein function indirectly [19,20]. In addition, in silico prediction methods, like molecular dynamics simulations, also use the principles of torsion angle changes to simulate protein movement and function [21]. 

This study calculates the backbone torsion angle fluctuation by analyzing the variation of backbone torsion angles from different NMR (nuclear magnetic resonance) models. NMR and X-ray crystallography are two different techniques used to study the structure of molecules, although they provide complementary information and are often used together to obtain a more complete picture [22]. NMR provides information on different time scales, ranging from picoseconds to seconds, making it a powerful tool for studying protein dynamics [23]. The long-time scales, in particular, enable the observation of slow conformational changes that would not be evident in short, instantaneous measurements, such as X-ray crystallography [22]. As a result, it offers a unique view into the overall flexibility and movement of protein structures [24,25]. The assumption that deposited ensembles are representative of these time scales is crucial. These ensembles can provide an aggregated view of possible protein conformations, which captures proteins’ inherent flexibility and adaptability. This breadth of structural information, combined with NMR data, allows for a more comprehensive picture of protein dynamics over time [14,26]. In essence, the combination of NMR measurements and ensemble representation allows for a more accurate prediction of protein dynamics and flexibility over long-time scales [27]. Given these considerations, this study has limitations in that it can provide predictions regarding the overall flexibility of each residue, irrespective of their local or global roles.

Several methods have been developed to predict backbone torsion angles. Angle predictions are useful in fold recognition [28,29] and fragment-based [30] or fragment-free structure prediction [31]. ANGLOR [32] utilizes support vector machines and neural networks for predicting the value of φ and ψ separately. TANGLE [33] uses a support vector regression method to predict backbone torsion angles (φ, ψ). Li et al. [34] predicted protein torsion angles by using four deep learning architectures, consisting of a deep neural network (DNN), a deep restricted Boltzmann machine (DRBN), a deep recurrent neural network (DRNN), and a deep recurrent restricted Boltzmann machine (DReRBM). In addition, Heffernan et al. [11] captured the nonlocal interactions and yielded the highest reported accuracy in angle prediction by using long short-term memory bidirectional recurrent neural networks. A good prediction of angle probability may provide significant information on structural flexibility and intrinsic protein disorder in extreme scenarios [33]. In recent times, there have been notable advancements in the field of protein structure prediction using deep learning techniques. Notably, AlphaFold [35], OmegaFold [36], and ESMFold [37] have exhibited impressive capabilities in predicting the three-dimensional (3D) structure of well-structured proteins. However, it is important to recognize that these methods excel primarily in predicting structured proteins [35]. On the contrary, the prediction of phi and psi angle fluctuations shows promise in assisting the prediction of unstructured or disordered protein structures.

However, to our knowledge, only one research project [16] presents work on backbone torsion angle fluctuation which is derived from the variation of backbone torsion angles. Since most proteins lack a known structure, identifying flexible regions, which may have functional significance, is a primary motivation for predicting torsion angle fluctuation based on protein sequence. Moreover, incorporating predicted torsion angles and flexibility as constraints can contribute to protein structure and disordered region predictions. Therefore, there is an urgent need to improve the extant method for predicting fluctuations in torsion angle from protein sequences. The only method we found was developed by Zhang et al. [16]. They only developed a neural network method for backbone torsion angle fluctuation based on sequence information. Their model achieved ten-fold cross-validated correlation coefficients of 0.59 and 0.60 and mean absolute errors (MAEs) of 22.7° and 24.3° for the angle fluctuation of φ and ψ, respectively. 

In this work, we developed a machine learning method [38], TAFPred, to predict backbone torsion angle fluctuation. Various features are directly extracted from protein sequences. A sliding window is used to include information from the neighbor residues. Furthermore, in TAFPred, we utilized a genetic algorithm (GA)-based feature selection method to extract several relevant features from the protein sequence. Finally, we trained an optimized light gradient boosting machine to predict the backbone torsion angle fluctuation. We believe this is the second work that presents a sequence-based prediction method for backbone torsion angle fluctuation. We anticipate that our work will contribute to further advancements in protein structure and protein disorder predictions.

## 2. Materials and Methods

**Figure 2:**
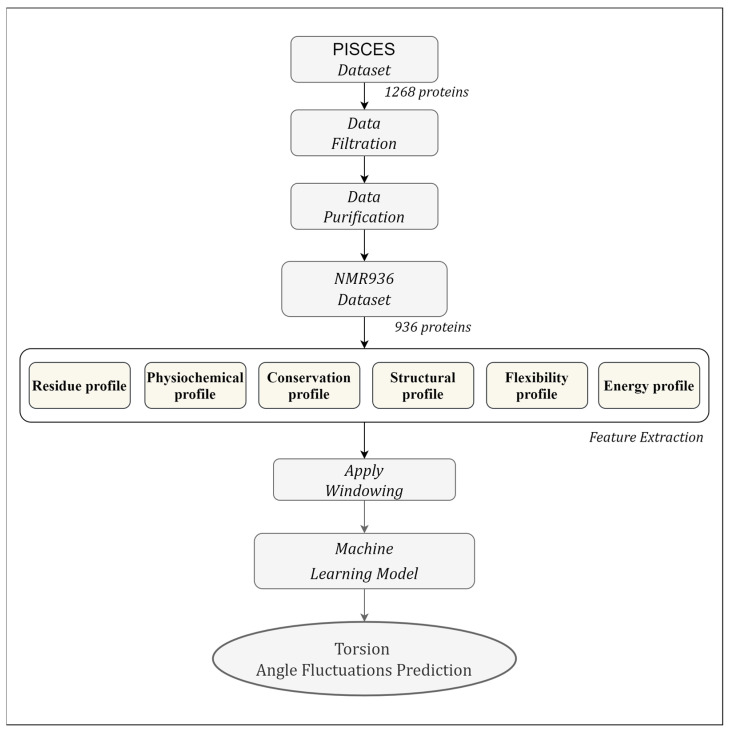
Illustration of the workflow of the torsion angle fluctuation predictions.

In this section, we provide a detailed description of the dataset used, the method employed for feature extraction, the evaluation metrics used to assess performance, the process of feature window selection, and, ultimately, the selected method for training the model. The workflow of the proposed TAFPred method is illustrated in Figure 2.

### 2.1. Dataset

We collected 1268 protein chains from the author [16]. These protein chains are determined using the nuclear magnetic resonance (NMR) method from the precompiled CulledPDB lists by PISCES using a sequence identity threshold of 25%. 997 protein chains are selected [16] by removing the chains with less than 5 NMR models, smaller than 25 amino acids, and consisting of nonstandard amino acid types. Finally, 936 protein chains are obtained by removing chains for which features could not be obtained (referred to as NMR936) [39]. The backbone torsion angle fluctuation is calculated by analyzing the variation of backbone torsion angles from different NMR models.

### 2.2. Feature Extraction

We extracted several relevant profiles from the protein sequences, i.e., the Residue profile, Conservation profile, Physiochemical profile, Structural profile, and Flexibility profile. Here, we briefly describe each of the profiles.

*Residue profile*. Twenty different numerical values are used to represent 20 standard amino acids (AA) types, yielding one feature per amino acid. The importance of this feature in solving bioinformatic problems has been shown in previous studies [40,41,42]. 

*Physiochemical profile*. In this work, five highly compact numeric patterns reflecting polarity, secondary structure, molecular volume, codon diversity, and electrostatic charge are extracted from [43] and used as features to represent the respective properties of each amino acid. 

*Conservation profile*. The protein sequence’s conservation profile is acquired through a normalized position-specific scoring matrix (PSSM) obtained from the DisPredict2 program [42]. The PSSM represents a matrix of L × 20 dimensions, where L denotes the protein sequence length. Higher scores in the PSSM indicate highly conserved positions, while scores near zero or that are negative indicate less conserved positions. The PSSM score was utilized to calculate monogram (MG) and bi-gram (BG) features. In terms of transition probabilities from one amino acid to another, the MG and BG properties can be used to characterize the portion of a protein sequence that can be conserved within a fold. From the DisPredict2 tool, we collect 1-D MG and 20-D BG characteristics.

*Structural profile*. Numerous biological problems have been solved using local structural features, such as the predicted secondary structure (SS) and accessible surface area (ASA) of amino acids. Here, the predicted ASA and SS probabilities for helix (H), coil (C), and beta-sheet (E) at the residue level are obtained from the DisPredict2 program. Moreover, we collect a separate set of SS probabilities for H, C, and E at the residue level from the BalancedSSP [44] program, as it provides a balanced prediction of these SS types. Thus, we extracted seven total structural properties (one ASA per amino acid and six predicted SS probabilities) as a structural profile of protein sequences.

*Flexibility profile*. Previous studies have demonstrated that an intrinsically disordered region (IDR) contains PTM sites, sorting signals, and playing an important role in regulating protein structures and functions [2,7,45]. In this study, we used a disorder predictor named DisPredict2 [42] to accurately predict the protein’s disordered regions and obtain the disorder probability as a feature. To further improve the feature quality, we obtained two predicted backbone angle fluctuations, dphi (ΔΦ) and dpsi (ΔΨ), the DAVAR program [16].

The energy profile by Iqbal and Hoque [42] proposed a novel method that uses contact energy and predicted relative solvent accessibility (RSA) to estimate the position-specific estimated energy (PSEE) of amino acid residues from sequence information alone. They showed that the PSEE could distinguish between a protein’s structured and unstructured or intrinsically disordered regions. We utilized the PSEE score per amino acid as a feature in our study since it has been empirically demonstrated to have the ability to address a number of biological issues.

### 2.3. Machine Learning Methods

We analyzed the performance of eight individual regression methods: (i) light gradient boosting machine regressor (LightGBM) [46]; (ii) extreme gradient boosting regressor (XGB) [47]; (iii) extra tree regressor (ET) [48]; (iv) decision tree regressor [49]; v) k-nearest neighbors regressor [49,50]; (vi) convolutional neural network (CNN) [49]; and long short-term memory (LSTM) [11]; and deep neural network (TabNet) [51]. The light gradient boosting machine regressor (LightGBM) performs better, as shown in the Results section.

### 2.4. Feature Selection Using Genetic Algorithm (GA)

**Figure 3:**
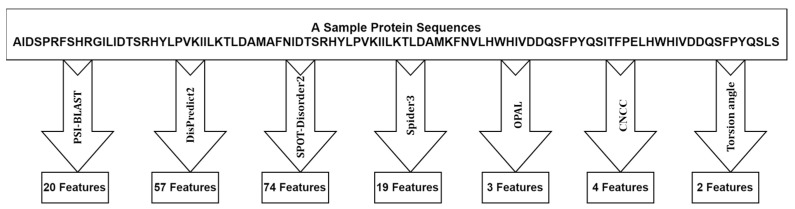
Feature extraction from different tools.

We collected a feature vector of 179 dimensions (Figure 3) from different tools during the feature extraction process. This feature vector is relatively large, and to mitigate dimensionality and enhance classification accuracy, we employed a genetic algorithm (GA), which belongs to the family of evolutionary algorithms, for feature selection. The GA algorithm was utilized to select relevant features that can contribute to improving the accuracy of classification. Further details regarding the feature selection approaches will be elaborated upon in the following sections.

A GA is a population-based stochastic search technique that mimics the natural process of evolution. It contains a population of chromosomes, each representing a possible solution to the problem under consideration. In general, a GA operates by initializing the population randomly and iteratively updating the population through various operators, including elitism, crossover, and mutation, to discover, prioritize, and recombine good building blocks in parent chromosomes and finally obtain fitter ones [52,53,54]. 

Encoding the solution of the problem under consideration in the form of chromosomes and computing the fitness of the chromosomes are two important steps in setting up the GA. The length of the chromosome space is equal to the length of the feature space. Moreover, to compute the chromosome’s fitness, we use the LightGBM algorithm [46,47]. LightGBM was chosen because of its fast execution time and reasonable performance compared to other machine learning classifiers. During feature selection, the values of LightGBM parameters, max_depth, eta, silent, objective, num_class, n_estimators, min_child_weight, subsample, scale_pos_weight, tree_method, and max_bin, were set to 6, 0.1, 1, ‘multi:softprob’, 2, 100, 5, 0.9, 3, ‘hist’, and 500, respectively, and the rest of the parameters were set to their default value. The values of the LightGBM parameters mentioned above were identified through the hit-and-trial approach. In our implementation, the objective fitness is defined as:

(1) $$obj_{fit}=1-MAE+PCC$$

### 2.5. Performance Evaluation

The performance evaluation of all the machine learning methods was conducted using a 10-fold cross-validation approach with the evaluation metric displayed in Table 1. We measure the performance of torsion angle fluctuation predictions by calculating the Pearson correlation coefficient (PCC) and mean absolute error (MAE) with the following equations:

## 3. Results

In this section, we first show the performance of different machine learning methods. Then, we present the performance of the best model with optimized hyperparameters. Next, we present the applied sliding window technique results to find the optimum window size. Finally, we compared the proposed method with the state-of-the-art method.

### 3.1. Comparison between Different Methods

We experimented with eight machine learning methods. The performance comparison of the individual regressors on the training dataset for phi angle fluctuation is shown in Table 2. Most of the methods perform better than the state-of-the-art method [16], except decision tree regressor. Table 2 further shows that the LightGBM is the best-performing regressor among the eight regressors implemented in our study regarding mean absolute value (MAE) and Pearson correlation coefficient (PCC). Moreover, LightGBM improves by 6.59% and 24.50% in terms of MAE and PCC, respectively, compared to the existing method.

Table 3 compares the individual regressors’ performance for psi angle fluctuations. Notably, the LightGBM regressor outperforms other methods, achieving an MAE of 0.127 and a PCC of 0.733. Furthermore, compared to the state-of-the-art method, the LightGBM Regressor demonstrates a significant improvement of 6.59% in MAE and 24.50% in PCC.

### 3.2. Hyperparameters Optimization

We optimized the LightGBM regressor parameters, learning_rate, estimators, max_depth, num_leaves, max_bin, feature_fraction, etc., to achieve the best 10-fold cross-validation performance and for sampling hyperparameters and pruning efficiently unpromising trials. We have used the custom objective function of [PCC+(1-MAE)] for optimization. The best values of the parameters, learning_rate, estimators, max_depth, num_leaves, max_bin, and feature_fraction, were found to be 0.014, 2561, 19, 380, 138, and 0.52, respectively.

### 3.3. Feature Window Selection

Here, we applied a widely used feature windowing technique to include the neighboring residue features. We examined a suitable sliding window size that determines the appropriate number of residues around a target residue that helps the model attain improved performance. We designed several models with different window sizes (ws) (1, 3, 5, and so on). We used the custom metric given in Equation (1) as the objective function to measure the performance of our proposed method. 

**Figure 4:**
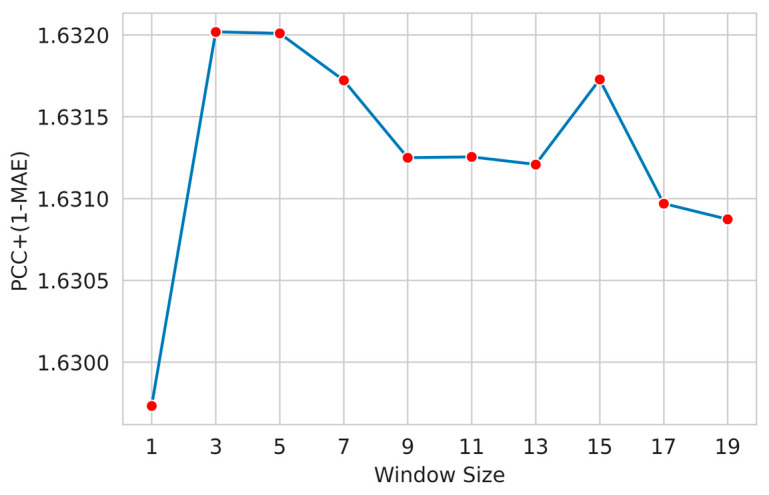
Selection of sliding window size with optimized LightGBM regressor (phi angle). Among the tested window sizes, it was found that a window size of 3 achieved the highest 1-MAE+PCC (mean absolute error + Pearson correlation coefficient) for the psi angle.

Figure 4 shows the performance of the optimized LightGBM regressor for different window sizes for the phi angle. The LightGBM regressor slightly improves window size 3, and the performance gradually decreases.

**Figure 5:**
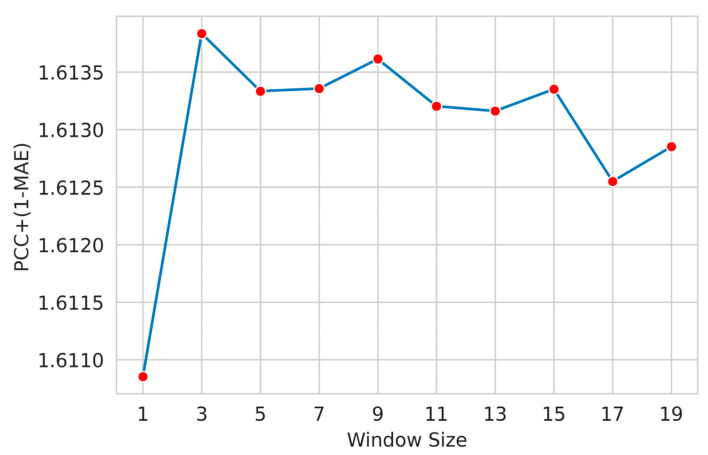
Selection of sliding window size with optimized LightGBM regressor (psi angle). Among the tested window sizes, it was found that a window size of 3 yielded the highest value of 1-MAE+PCC for the psi angle.

Figure 5 shows the performance of the optimized LightGBM regressor for different widow sizes for psi angle. The LightGBM regressor performance improves for a window size of 3, and then the performance gradually decreases. For this reason, we selected a window size of 3 to train the final model.

### 3.4. Comparison with the State-of-the-Art Method

Here, we compare the performance of the proposed method, TAFPred, with an existing state-of-the-art method [16] proposed by Zhang et al. Table 4 shows that our proposed method improves by 10.08% in MAE and 24.83% in PCC in the phi angle compared to the state-of-the-art method [16].

Table 5 shows that our proposed method improves by 9.93% in MAE and 22.37% in PCC in psi angle compared to the state-of-the-art method. Our proposed method significantly outperforms the existing state-of-the-art method and can more accurately predict the protein’s backbone torsion angle fluctuations.

## 4. Discussion

In this section, we explore diverse characteristics associated with the distribution of torsion angle fluctuation. We examine the correlation between Δφ and Δψ, as well as the connection between torsion-angle fluctuation and disordered regions, utilizing our newly generated dataset.

### 4.1. The Distribution of Torsion-Angle Fluctuation

**Figure 6:**
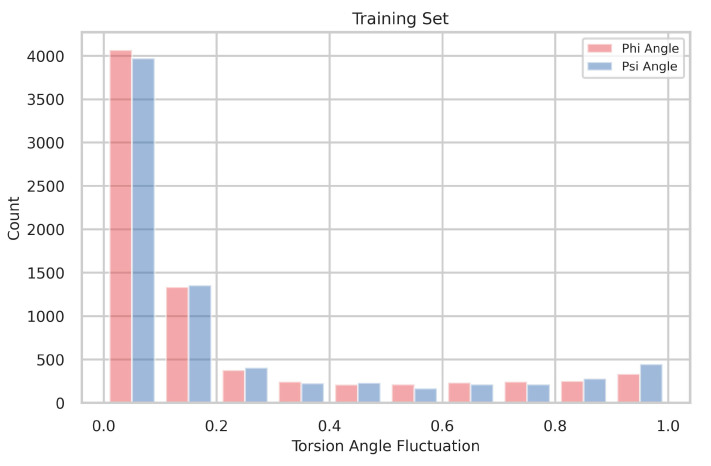
The torsion-angle fluctuation is depicted in its distribution, with the data points divided into 10 bins. The fluctuations of the phi and psi angles are visually represented using red and green colors, respectively.

Figure 6 displays the distribution of torsion-angle fluctuation, with the dataset divided into 10 bins. The distributions are nonuniform, and most residues exhibit angle fluctuations below 0.2. This observation indicates that a limited presence of flexible residues characterizes stable protein structures.

### 4.2. Relationship between Δφ and Δψ 

**Figure 7:**
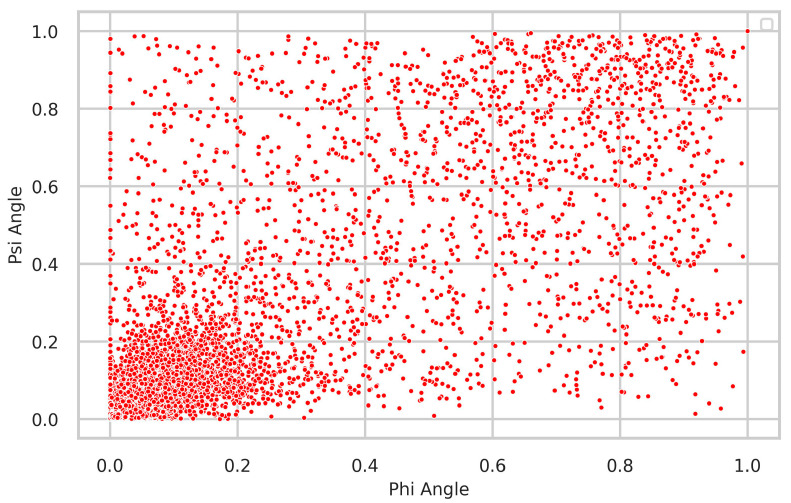
The relationship between Δφ and Δψ is shown in the figure, revealing that the majority of residues exhibit small fluctuations below 0.2.

We further examined the relationship between the Δφ and Δψ angles (Figure 7), which represent the fluctuation of neighboring rotational angles in the protein backbone for the same residue. A chemical bond linkage correlates these angles, as it is impossible to alter one torsion angle without affecting the other. As expected, a pronounced and statistically significant correlation was observed between them. In line with expectations, most residues demonstrated minimal fluctuations below 0.2. 

### 4.3. Relationship between Torsion-Angle Fluctuation and Disordered Regions

**Figure 8:**
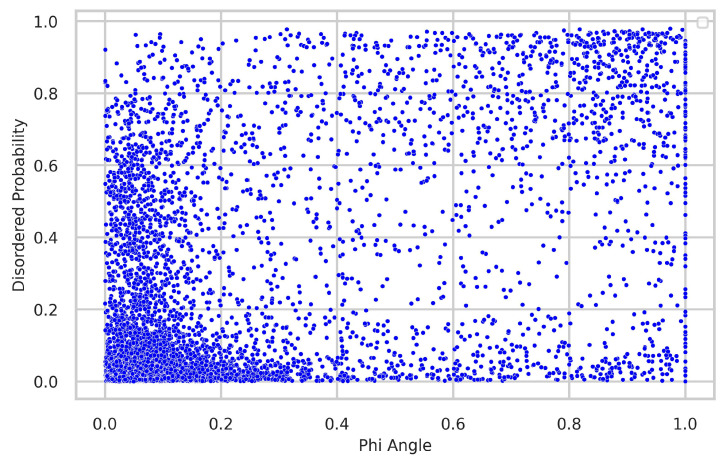
Relationship between torsion-angle fluctuation in the phi angle and disordered regions. The disordered probability was obtained from the SPOT-Disordered2 tool. The figure illustrates that regions with low disordered probability exhibit correspondingly low fluctuations in the phi angle, and conversely, regions with high disordered probability show higher fluctuations in the phi angle.

**Figure 9:**
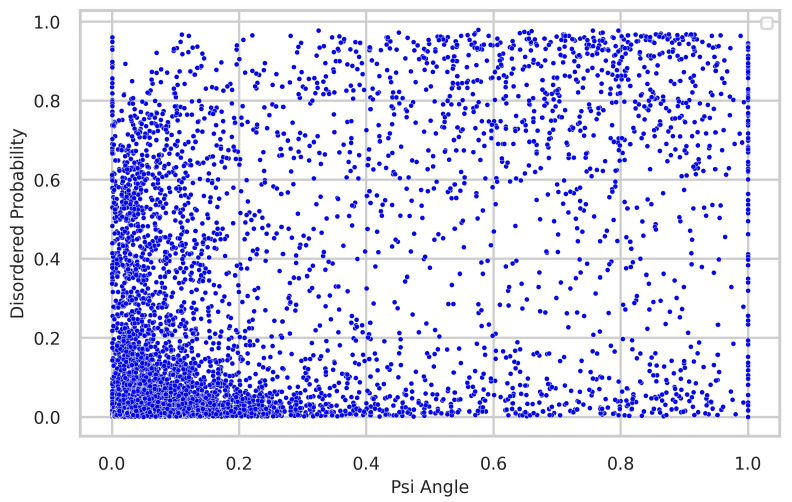
Correlation between torsion-angle fluctuation in the psi angle and the presence of disordered regions. The disordered probability was obtained through the utilization of the SPOT-Disordered2 tool. The figure clearly illustrates that regions with low disordered probability exhibit lower fluctuations in the psi angle, while regions with high disordered probability tend to have higher fluctuations in the psi angle.

We thoroughly investigated the connection between torsion-angle fluctuation and disordered regions. To gather disordered probability data, we utilized the SPOT-Disordered2 method. The figures provide clear evidence of the close relationship between phi and psi angle fluctuations and the presence of disordered regions. In the majority of samples, regions with low fluctuations exhibit a low disordered probability, while regions with higher fluctuations display a higher disordered probability, as illustrated in Figure 8 and Figure 9.

## 5. Conclusions

This study explored eight machine learning methods, including a recently published Deep Neural Network (TabNet) [51], to determine their effectiveness. Among these methods, the light gradient boosting machine regressor (LightGBM) emerged as the best performer in terms of MAE and PCC. To optimize LightGBM regressor, we used state-of-the-art sampling and pruning algorithms for hyperparameter tuning. Moreover, a custom objective function is used for optimization, and a sliding window technique is used to extract more information from the neighbor residues for improved performance. Our proposed method, TAFPred, shows an average improvement of 15.54% and 13.96% in both metrics (MAE and PCC) on phi and psi angles, respectively, compared to the state-of-the-art method [16]. In the future, we also plan to investigate the impact of torsion angle fluctuation in disorder proteins. We firmly believe the developed method will be helpful to the researcher in protein structure prediction and disordered prediction.

## Figures and Tables

**Table 1:** Performance evaluation metrics.

Name of Metric	Definition
Pearson Correlation Coefficient (PCC) =	$\frac{\sum_{i=1}^{N}\left(x_{i}-\overset{-}{x})(y_{i}-\overset{-}{y}\right)}{\sqrt{[\sum_{i=1}^{N}(x_{i}-\overset{-}{x}{)}^{2}][\sum_{i=1}^{N}\left(y_{i}-\overset{-}{y}{)}^{2}\right]}}$
Mean Absolute Error (MAE) =	$\frac{1}{N}\sum_{i=1}^{N}\left|x_{i}-y_{i}\right|$

Here, *x*_*i*_ is the predicted torsion angle fluctuation, *y*_*i*_ is the native torsion angle fluctuation for the *i* residue in the sequence, and $\overset{-}{x}$ and $\overset{-}{y}$ are their corresponding sample means.

**Table 2:** Results from different machine learning methods (phi angle).

Methods/Metric	MAE	PCC	MAE (% imp.)	PCC (% imp.)	Average (% imp.)
**State-of-the-art Method** [16]	0.126	0.598	-	-	-
Extra Trees Regressor	0.122	0.741	3.57%	23.88%	13.73%
XGB Regressor	0.123	0.727	2.67%	21.57%	12.12%
KNN Regressor	0.129	0.681	−2.30%	13.89%	5.79%
Decision Tree Regressor	0.167	0.527	−24.38%	−11.84%	−18.11%
LSTM	0.125	0.678	1.13%	13.35%	7.24%
CNN	0.166	0.608	−24.21%	1.68%	−11.27%
Tabnet	0.117	0.736	7.26%	23.09%	15.18%
**LightGBM Regressor**	**0.118**	**0.745**	**6.59%**	**24.50%**	**15.54%**

Best score values are **boldfaced**. Here, ‘imp.’ stands for improvement. The ‘% imp.’ represents the improvement in percentage achieved by TAFPred compared to the state-of-the-art method. Likewise, the ‘Average (% imp.)’ represents the average percentage improvement achieved by TAFPred for both MAE and PCC. Additionally, ‘(-)’ denotes that the % imp. or (Average % imp.) cannot be calculated.

**Table 3:** Results from different machine learning methods (psi angle).

Methods/Metric	MAE	PCC	MAE (% imp.)	PCC (% imp.)	Average (% imp.)
**State-of-the-art Method** [16]	0.135	0.602	-	-	-
Extra Trees Regressor	0.131	0.729	2.77%	21.10%	11.94%
XGB Regressor	0.132	0.715	2.22%	18.73%	10.48%
KNN Regressor	0.139	0.670	−2.63%	11.24%	4.31%
Decision Tree Regressor	0.179	0.511	−24.65%	−15.11%	−19.88%
LSTM	0.132	0.665	2.29%	10.48%	6.38%
CNN	0.144	0.702	−6.46%	16.61%	5.07%
Tabnet	0.126	0.724	7.24%	20.28%	13.76%
**LightGBM Regressor**	**0.127**	**0.733**	**6.09%**	**21.84%**	**13.96%**

Best score values are **boldfaced**. Here, ‘imp.’ stands for improvement. The ‘% imp.’ represents the improvement in percentage achieved by TAFPred compared to the state-of-the-art method. Likewise, the ‘Average (% imp.)’ represents the average percentage improvement achieved by TAFPred for both MAE and PCC. Additionally, ‘(-)’ denotes that the % imp. or (Average % imp.) cannot be calculated.

**Table 4:** CV Results with optimized LightGBM regressor with a sliding windows size of 3 (phi angle).

Methods/Metric	MAE	PCC	MAE (% imp.)	PCC (% imp.)	Average (% imp.)
**State-of-the-art Method** [16]	0.126	0.598	-	-	-
**TAFPred**	**0.114**	**0.746**	**10.08%**	**24.83%**	**17.45%**

Best score values are **boldfaced**. Here, ‘imp.’ stands for improvement. The ‘% imp.’ represents the improvement in percentage achieved by TAFPred compared to the state-of-the-art method. Likewise, the ‘Average (% imp.)’ represents the average percentage improvement achieved by TAFPred for both MAE and PCC. Additionally, ‘(-)’ denotes that the % imp. or (Average % imp.) cannot be calculated.

**Table 5:** Cross-validation results with a sliding windows size of 3 (psi angle).

Methods/Metric	MAE	PCC	MAE (% imp.)	PCC (% imp.)	Average (% imp.)
**State-of-the-art Method** [16]	0.135	0.602	-	-	-
**TAFPred**	**0.123**	**0.737**	**9.93%**	**22.37%**	**16.15%**

Best score values are **boldfaced**. Here, ‘imp.’ stands for improvement. The ‘% imp.’ represents the improvement in percentage achieved by TAFPred compared to the state-of-the-art [16] method. Likewise, the ‘Average (% imp.)’ represents the average percentage improvement achieved by TAFPred for both MAE and PCC. Additionally, ‘(-)’ denotes that the % imp. or (Average % imp.) cannot be calculated.

## Data Availability

The code and data related to the development of TAFPred can be found here: https://github.com/wasicse/TAFPred (accessed on 1 May 2023). The TAFPred webserver is available at https://bmll.cs.uno.edu (accessed on 1 May 2023).
